# Prognostic value of hematological markers for anastomotic leakage diagnosed after postoperative day 5 following laparoscopic surgery for colorectal cancer

**DOI:** 10.1186/s12893-026-03955-4

**Published:** 2026-06-18

**Authors:** Zhengwei Wen, Zhengqi Wen, Ruize Zhou, Xihong Liu, Jinjin Luo, Shipan Fan, Jibang Peng, Yahan Zhang, Xueqin Wang

**Affiliations:** 1 Department of Surgical Oncology, The First Affiliated Hospital of Kunming Medical University, No. 295 Xichang Road, Wuhua District, Kunming, Yunnan 650032 China; 2 General Surgery Department, Mengzi People’s Hospital, Mengzi City, Yunnan 661100 China

**Keywords:** Colorectal cancer, Anastomotic leakage, HCT-ALB, Neutrophil-to-albumin ratio, Neutrophil-to-lymphocyte ratio

## Abstract

**Background:**

Anastomotic leakage (AL) is a common complication following colorectal cancer surgery, yet accurate and unified diagnostic criteria remain lacking.

**Methods:**

This retrospective study included 360 patients who underwent laparoscopic colorectal resection with one-stage anastomosis at the First Affiliated Hospital of Kunming Medical University between October 2019 to December 2023. Hematocrit-to-albumin difference (HCT-ALB), neutrophil-to-albumin ratio (NAR), and neutrophil-to-lymphocyte ratio (NLR) on postoperative days (POD) 1, 3, and 5 were compared between patients with and without AL. This study specifically aimed to evaluate the predictive performance of these markers for AL occurring after POD 5.

**Results:**

Compared to the non-AL group, the AL group exhibited significantly higher HCT-ALB, NAR, and NLR values on POD 3 and 5 (*P* < 0.05), except for NLR on POD 1. Multivariate analysis identified rectal tumor location, HCT-ALB and NLR on POD 3, and NAR and NLR on POD 5 as associated factors of AL, while preoperative ALB, HCT-ALB on POD1 and POD5, and NAR on POD1 and POD3 showed weaker associations (*P* > 0.05). The best predictors were NAR on POD 5 (AUC = 0.910, cut-off = 2.09) and NLR on POD 5 (AUC = 0.885, cut-off = 7.19). Due to the exploratory nature of this study and the potential for collinearity between these inflammatory markers, these results should be interpreted with caution.

**Conclusion:**

Tumor location (rectum), HCT-ALB and NLR on POD 3, and NAR and NLR on POD 5 may serve as reliable indicators for AL, thus enabling early identification and timely intervention to improve postoperative outcomes after colorectal surgery.

## Background

Colorectal cancer (CRC) is a common malignancy with one of the highest incidence and mortality rates worldwide. According to statistics, the global incidence of colorectal cancer in 2022 was approximately 1,926,000, with an incidence of 9.6%; the number of deaths was approximately 904,000, with a mortality rate of 9.3% [1]. Surgery is the main curative modality for CRC with laparoscopic surgical techniques representing the standard approach [2]. Surgery for CRC inevitably leads to complications with anastomotic leakage (AL) the most common and yielding the worst consequences. AL may result in the need for reoperation, delayed hospital discharge, an elevated risk of local recurrence, and increased complication, morbidity and mortality rates. The incidence of postoperative AL in CRC ranges from 2.8% to 30%, with 75% of all cases involving rectal anastomoses. The mortality and morbidity rates of AL after rectal cancer surgery range from 2% to 16.4% and 20% to 35%, respectively [3]. Consequently, the early detection and prediction of AL and timely intervention are crucial if we are to improve patient prognoses and minimize inflammatory responses.

Most previous studies on AL focused on risk factors and the retrospective analyses of rectal resection cases. These studies typically assessed risk through an integrated evaluation of clinical indicators, including vital signs, symptomatology, abdominal drainage characteristics, initial defecation timing, computed tomography (CT) imaging, serological markers (e.g., C-reactive protein [CRP] and procalcitonin), additional cytokines, biomarkers, and surgical re-examination, when necessary [4]. CT, the preferred imaging modality for diagnosing AL, presents challenges because of its reliance on ionizing radiation. Furthermore, the use of CT is particularly difficult for post-operative patients with limited mobility. Furthermore, the sensitivity and specificity of CT for the detection of AL are suboptimal, rendering this technique unsuitable for routine postoperative assessments. In contrast, laboratory markers are accessible, cost-effective, non-invasive and are increasingly being utilized for early postoperative AL prognostication studies [5]. However, because AL is a complex systemic process, a single laboratory index will have poor prediction accuracy.

Recent studies have emphasized the importance of hematological indicators for systemic inflammation, including the differences between hematocrit (HCT) and serum albumin (ALB) level (HCT-ALB), neutrophil percentage-to-albumin ratio (NAR), and neutrophil-to-lymphocyte ratio (NLR). These markers provide a nuanced evaluation of a patient’s inflammatory status, infection severity, and prognostic implications, thus surpassing conventional markers in their capacity to represent systemic health [6–8]. Accordingly, these markers exhibit substantial diagnostic and prognostic potential for AL. In the present study, we aimed to identify a more accurate predictor or early diagnostic tool for AL that would complement existing indices, thus providing clinicians with valuable references and improving the accuracy of AL prognoses following CRC resection.

## Methods

### Material

#### Data collection

We collated clinical data from patients who underwent colorectal surgery at the Department of Surgical Oncology at the First Affiliated Hospital of Kunming Medical University, between October 2019 and December 2023. This study was conducted in accordance with the guidelines of the Declaration of Helsinki and approved by the First Affiliated Hospital of Kunming Medical University Medical Ethics Committee (Ethics Batch Number: (2023) LUN Review L No. 201). Informed consent was obtained from all study participants.

Between October 2019 and December 2023, 1309 patients underwent CRC surgery at our center; of these, 60 (4.58%) developed AL. We excluded 949 patients according to strict inclusion and exclusion criteria and 20 patients with AL. The 20 patients with AL were excluded from the final analysis because their AL was diagnosed on or before postoperative day (POD) 5. This exclusion was implemented to ensure that the biomarker evaluations on POD 1, 3, and 5 strictly preceded the clinical manifestation of the complication, thereby avoiding causal inversion. Consequently, the study enrolled 360 patients categorized into two cohorts based on AL during their hospital stay and monitoring period. The AL cohort comprised 40 patients and the non-AL cohort included 320 patients.

#### Inclusion and exclusion criteria

The inclusion criteria were as follows: (a) patient underwent laparoscopic resection of a tumor followed by immediate anastomosis and CRC was pathologically verified, (b) availability of complete preoperative and postoperative peripheral blood tests results (days 1, 3, and 5), including neutrophil and lymphocyte counts, lymphocyte percentage, HCT, and ALB; and (c) availability of comprehensive baseline demographic and clinical data.

The exclusion criteria were as follows: (a) patient underwent open or conversion surgery or simultaneous multi-organ resections; (b) a history of abdominal surgery within three months preceding definitive CRC surgery; (c) patient underwent Hartmann’s or Miles’ procedures, emergency surgery, or palliative surgery; and (d) infectious complications in addition to AL, including infections in the surgical incision, lungs or urinary tract; (e) Patients with early-onset AL diagnosed on or before POD 5 were excluded to ensure that biomarker evaluations on POD 1, 3, and 5 strictly preceded the clinical manifestation of the complication.

### AL diagnosis

According to the widely adopted Clavien-Dindo classification (2004) [9] and the consensus definition and grading system by the International Study Group of Rectal Cancer (2010) [10, 11], AL was diagnosed when patients satisfied at least two of the following criteria: (1) recurrent postoperative fevers exceeding 38 °C, (2) persistent abdominal pain, distension, or signs of peritonitis, accompanied by gas, pus, or feculent material from the abdominal drain, (3) sustained elevated levels of inflammatory markers, including leukocytes, neutrophils, and CRP, (4) CT imaging showed intra-abdominal fluid collection and pneumoperitoneum close to the site of anastomosis, with gastrointestinal angiography indicating contrast leakage, and (5) the detection of anastomotic disruption upon rectal examination.

### Statistical analysis

Statistical analyses were performed using SPSS version 27.00 software. Differences were considered statistically significant when *P* < 0.05. Normality tests were performed on all data, and the independent samples t-test was used to compare differences between groups for measurements that conformed to the normal distribution. The rank sum test was used to compare differences between groups for measurements that were not normally distributed. The chi-squared test was used to compare differences in count data between groups. Significantly different indicators in the AL and non-AL groups were subjected to receiver operating characteristic (ROC) curve analysis to investigate their predictive values for postoperative AL. The sensitivity, specificity and critical value were calculated using the Jordan index. Binary logistic regression was used for multi-factorial analysis to identify independent risk factors for AL. The probability of these risk factors jointly predicting AL was calculated and ROC curves were plotted.

## Results

This study included 360 cases, with 40 (11.11%) diagnosed with AL and 320 (88.89%) classified as non-AL. The cohort consisted of 219 males (60.83%) and 141 females (39.17%). In the AL group, 33 patients (82.5%) had rectal involvement and seven (17.5%) had colonic involvement. In contrast, the non-AL group featured 163 rectal cases (50.94%) and 157 colonic cases (49.06%). The AL group was 75% male (*n* = 30) and 25% female (*n* = 10). The non-AL group featured 189 males (59.1%) and 131 females (40.9%) (Table 1). Notably, the clinical or radiological diagnosis of AL in all 40 patients occurred exclusively after POD 5, with a median diagnosis time of POD 8 (range: POD 6–14). No patients with early-onset AL (diagnosed on or before POD 5) were included in the final analysis.

**Table 1:** Comparison of clinical data between the two groups of patients

General information	AL group (*n* = 40)	Non-AL group (*n* = 320)	t/x²	*P*
Sex			3.790	0.052
Men	30 (75%)	189 (59.1%)		
Women	10 (25%)	131 (40.9%)		
Age	68 (58, 72)	65 (54, 65)	−1.279	0.201
BMI (kg/m2)	23.21 ± 4.01	22.67 ± 3.22	0.968	0.333
Hypertension			0.846	0.358
Yes	18 (45%)	120 (37.5%)		
No	22 (55%)	200 (62.5%)		
Diabetes mellitus			0.057	0.811
Yes	8 (20%)	59 (18.4%)		
No	32 (80%)	261 (81.6%)		
History of smoking			0.706	0.401
Yes	13 (32.5%)	84 (26.3%)		
No	27 (67.5%)	236 (73.7%)		
History of drinking			0.277	0.599
Yes	7 (17.5%)	46 (14.4%)		
No	33 (82.5%)	274 (85.6%)		
Tumor site			14.281	*
Rectum	33 (82.5%)	163 (50.94%)		
Colon	7 (17.5%)	157 (49.06%)		
TNM by stages			2.877	0.090
I + II	28 (70%)	179 (55.94%)		
III + IV	12 (30%)	141 (44.06%)		
Preoperative percentage of neutrophils (%)	63.16 ± 11.495	61.44 ± 10.148	0.993	0.321
Preoperative lymphocyte percentage (%)	26.64 ± 10.12	28.33 ± 9.425	0.507	0.288
Preoperative neutrophil count (×109/L)	3.88 (2.87, 4.70)	3.47 (2.78, 4.43)	−1.010	0.312
Preoperative lymphocyte count (×109/L)	1.56 (1.25, 1.98)	1.59 (1.23, 2.03)	−0.408	0.683
Preoperative HCT (%)	4.05 (1.40, 6.78)	3.90 (1.80, 6.48)	−0.015	0.988
Preoperative ALB (g/L)	40.30 (35.85, 44.10)	42.80 (39.70, 45.10)	−2.540	0.011

*AL* anastomotic leak, *BMI* body mass index, *TNM stage* American Joint Committee on Cancer (AJCC) eighth edition TNM stage, *HCT* hematocrit, *ALB* serum albumin. *P < 0.001

No statistically significant differences were observed between the AL and non-AL groups after laparoscopic CRC surgery (*P* > 0.05) in terms of gender, age, body mass index (BMI), hypertension, diabetes mellitus, smoking history, alcohol consumption history, TNM stage, preoperative peripheral blood metrics including neutrophil and lymphocyte percentages, neutrophil and lymphocyte counts, and HCT. In contrast, tumor locations and preoperative serum ALB levels differed significantly between the two groups (*P* < 0.05). A significantly greater proportion of tumors in the AL group were detected in the rectum than in the colon (*P* < 0.05). In addition, preoperative ALB levels were significantly lower in the AL group than in the non-AL group (*P* < 0.05) (Table 1).

A comparison of the pre- and postoperative HCT-ALB, NAR, and NLR revealed that NAR and HCT-ALB values on postoperative days (PODs) 1, 3, and 5 and NLR on POD3 and 5 were significantly greater in the AL group than in the non-AL group (*P* < 0.05) (Table 2).

**Table 2:** Comparison of HCT-ALB, NAR and NLR in the two groups of patients

Factor	AL group (*n* = 40)	Non-AL group (*n* = 320)	t/z	*P*
HCT-ALB				
Preoperative	4.05 (1.40, 6.78)	3.90 (1.80, 6.48)	−0.015	0.988
POD1	6.50 (3.65, 9.35)	4.20 (1.90, 7.10)	−3.276	*
POD3	6.35 (2.55, 10.75)	3.90 (1.80, 6.90)	−2.586	0.010
POD5	5.25 (2.28, 9.10)	3.90 (1.82, 6.98)	−1.989	0.047
NAR				
Preoperative	1.56 (1.32, 1.95)	1.43 (1.26, 1.65)	−2.023	0.043
POD1	2.62 (2.32, 2.96)	2.39 (2.19, 2.64)	−3.200	*
POD3	2.46 (2.11, 2.78)	2.06 (1.88, 2.35)	−4.607	*
POD5	2.64 (2.25, 2.96)	1.85 (1.64, 2.12)	−8.465	*
NLR				
Preoperative	2.53 (1.55, 3.73)	2.12 (1.64, 3.06)	−1.186	0.236
POD1	9.43 (6.40, 13.15)	8.98 (5.60, 14.60)	−0.411	0.681
POD3	10.16 (7.44, 16.26)	6.60 (4.52, 10.52)	−3.918	*
POD5	12.32 (7.57, 19.42)	4.20 (2.93, 6.13)	−7.951	*

*AL* anastomotic leakage, *POD* postoperative day, *HCT-ALB*: difference between hematocrit (%) and serum albumin (g/L), *NAR* ratio of neutrophil percentage (%) to serum albumin (g/L), *NLR* ratio of neutrophil count (×10^9^/L) to lymphocyte count (×10^9^/L). *P < 0.001

Postoperative AL was set as the dependent variable. For multi-factorial analysis, we included variables from previous studies that identified statistical significance in two-group comparisons: preoperative NAR, HCT-ALB, and NLR [7, 8, 12]. Specifically, we considered HCT-ALB and NAR measurements on POD1, POD3, and POD5, and NLR measurements on POD3 and POD5. In addition, we incorporated general preoperative data, including tumor location and preoperative ALB level. A binary logistic regression model was used for multi-factorial analysis. Analysis revealed that tumor location in the rectum, HCT-ALB, NLR on POD3, and NAR and NLR on POD5, were independent predictors of AL after laparoscopic surgery for CRC (Table 3). In contrast, preoperative ALB levels, HCT-ALB on POD1 and POD5, and preoperative, POD1, and POD3 NAR were not risk factors for postoperative AL (*P* > 0.05) (Table 3).

**Table 3:** Multifactorial logistic regression analysis of risk factors for AL after laparoscopic radical colorectal surgery

Factor	B	SE	Wald	*P*	OR	95% CI of the OR	
						Lower limit	Superior limit
Tumor site	−2.004	0.64	9.801	0.002	0.135	0.038	0.473
Preoperative ALB (g/L)	−0.093	0.072	1.697	0.193	0.911	0.791	1.048
HCT-ALB							
POD1	−0.001	0.063	0.001	0.985	0.999	0.883	1.13
POD3	0.218	0.082	7.103	0.008	1.243	1.059	1.459
POD5	−0.14	0.076	3.421	0.064	0.87	0.75	1.008
NAR							
Preoperative	−0.229	0.783	0.086	0.77	0.795	0.171	3.691
POD1	−0.002	0.85	0.001	0.998	0.998	0.189	5.277
POD3	0.469	0.758	0.383	0.536	1.598	0.362	7.053
POD5	3.107	0.787	15.6	*	22.348	4.783	104.424
NLR							
POD3	−0.142	0.06	5.674	0.017	0.868	0.772	0.975
POD5	0.245	0.064	14.847	*	1.278	1.128	1.448
Constant scale	−6.576	4.634	2.014	0.156	0.001		

*ALB* serum albumin (g/L), *HCT-ALB* difference between hematocrit (%) and serum albumin (g/L), *POD* postoperative day, *NAR* ratio of neutrophil% (%) to serum albumin (g/L), *NLR* ratio of neutrophil count (×10^9^/L) to lymphocyte count (×10^9^/L), *AL* anastomotic leak, *CI* confidence interval, *OR* overall risk. *P < 0.001

**Fig. 1:**
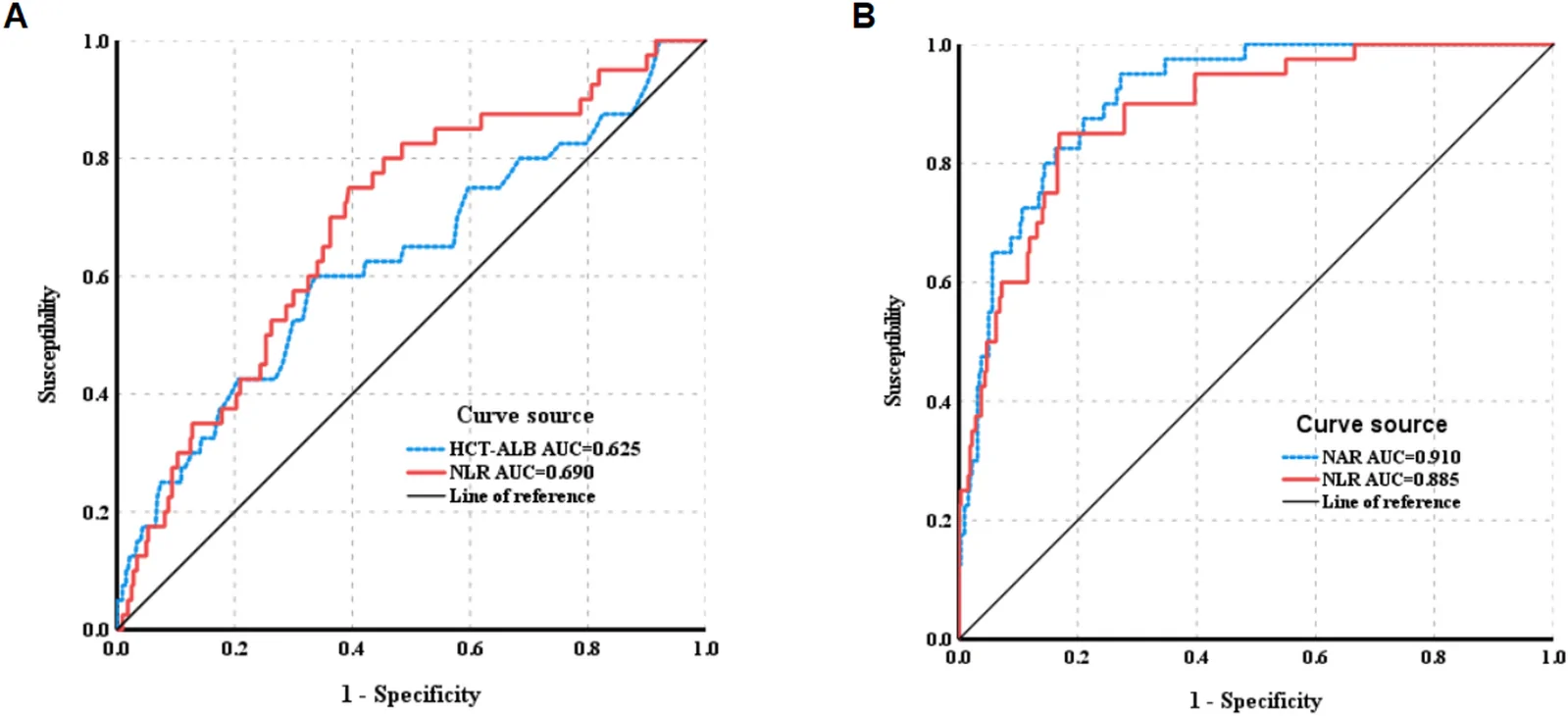
ROC curve analysis of HCT-ALB, NAR, and NLR predictions for AL. **A** ROC curves showing HCT-ALB and NLR predictions for AL on postoperative day 3: HCT-ALB (AUC = 0.625, cut-off = 5.8, sensitivity = 60%, specificity = 66.9%); NLR (AUC = 0.690, cut-off = 7.75, sensitivity = 75%, specificity = 66.6%); **B** ROC curves showing NAR and NLR predictions for AL on postoperative day 5: NAR (AUC = 0.910, cut-off = 2.09, sensitivity = 95%, specificity = 72.8%); NLR (AUC = 0.885, cut-off = 7.19, sensitivity = 85%, specificity = 83.1%). HCT-ALB: difference between hematocrit (%) and serum albumin (g/L); POD: postoperative day; NAR: ratio of neutrophil percentage (%) to serum albumin (g/L); NLR: ratio of neutrophil count (×109/L) to lymphocyte count (×109/L); AL: anastomotic leakage. *P < 0.001

Next, we performed ROC curve analyses to evaluate the predictive performance of HCT-ALB and NLR on POD3, and NAR and NLR on POD5 for AL. The area under the curve (AUC) for HCT-ALB on POD3 was 0.625 (95% confidence interval [CI]: 0.525–0.725), with a 5.8 threshold, 60% sensitivity, 66.9% specificity, 18.18% positive predictive value (PPV), and 92.98% negative predictive value (NPV). On the same day, the NLR predicted AL with a 0.690 AUC (95% CI: 0.605–0.774), 7.75 threshold, 75% sensitivity, 66.6% specificity, 19.61% PPV, and 95.16% NPV. On POD5, the AUC for NAR was 0.910 (95% CI: 0.872–0.949), with a 2.09 threshold, 95% sensitivity, 72.8% specificity, 30.15% PPV, and 99.14% NPV. For the same timepoint, NLR predicted AL with a 0.885 AUC (95% CI: 0.835–0.936), 7.19 threshold, 85% sensitivity, 83.1% specificity, 38.63% PPV, and 97.79% NPV (Fig. 1; Table 4).

**Table 4:** Comparison of ROC curves for HCT-ALB and NLR on POD3, and NAR and NLR in predicting AL on POD5

Factor	AUC (95% CI)	Critical value	Sensibility	Specificity	Positive predictive value	Negative predictive value	*P*
POD3							
HCT-ALB	0.625 (0.525–0.725)	5.8	60%	66.9%	18.18%	92.98%	0.010
NLR	0.690 (0.605–0.774)	7.75	75%	60.6%	19.61%	95.16%	*
POD5							
NAR	0.910 (0.872–0.949)	2.09	95%	72.8%	30.15%	99.14%	*
NLR	0.885 (0.835–0.936)	7.19	85%	83.1%	38.63%	97.79%	*

*HCT-ALB* difference between hematocrit (%) and serum albumin (g/L), *POD* postoperative day, *NAR* ratio of neutrophil percentage (%) to serum albumin (g/L), *NLR* ratio of neutrophil count (×10^9^/L) to lymphocyte count (×10^9^/L), *AL* anastomotic leakage. *P < 0.001

## Discussion

Despite advancements in science, technology and surgical techniques that reduce the incidence of AL, AL remains a significant challenge for gastrointestinal surgeons. AL significantly exacerbates the postoperative recovery and prognosis of patients with CRC and increases the risk of postoperative recurrence [13]. Timely detection and accurate diagnosis of AL, coupled with effective and timely interventions, are crucial if we are to enhance prognoses and mitigate severe adverse outcomes [14]. Investigating the risk factors and early diagnostic and prognostic biomarkers of AL is imperative if we are to improve clinical outcomes. In this study, we demonstrated that low preoperative ALB levels can predict AL after CRC surgery. Low preoperative ALB levels, indicative of a poor nutritional status and an increased risk of infection, correlated with AL, while HCT-ALB, NAR, and NLR reflected systemic inflammation and metabolic changes, thus enhancing their predictive value for AL [15–16]. We also identified the tumor site (rectum), HCT-ALB level, NLR on POD3, and NAR and NLR on POD5, as potential independent risk factors for the development of AL after laparoscopic surgery for CRC. Compared to existing studies on NLR and NAR in postoperative complications, our study uniquely evaluated these markers alongside HCT-ALB across multiple postoperative days (POD1, POD3, POD5) to predict AL specifically after laparoscopic CRC surgery. This longitudinal approach revealed dynamic changes in inflammatory markers, with NAR on POD5 demonstrating superior predictive accuracy (AUC = 0.910, cut-off = 2.09) compared to single-timepoint analyses or other biomarkers like CRP, which is limited by non-specificity and cost. These findings provide clinicians with precise and time-specific thresholds for the early detection of AL. To rule out causal inversion, the chronological sequencing between biomarker kinetics and AL onset must be highlighted. In this study, all AL events were confirmed strictly after POD 5. Since blood sampling on POD 1, 3, and 5 preceded overt clinical symptoms, the high predictive performance (especially POD 5 NAR) reflects true subclinical systemic inflammation rather than secondary consequences of established leakage.

Tumor location is a known to be a significant risk factor for AL [17]. If a tumor is situated in the lower intestinal segment, then the surgical field becomes constrained, thus resulting in limited operating space, increased surgical difficulty, and potential collateral vessel injury. In contrast, a low anastomosis weakens the protective effect of the serosal layer, impairs blood supply to the lower rectum, and may cause conditional sphincter contraction post-operatively, thus potentially leading to anastomotic stricture, an elevated risk of AL, and other complications [18–19]. It is important to emphasize that our findings are specific to AL diagnosed after POD 5. By excluding early-onset AL cases, we ensured that the measured biomarkers reflect subclinical systemic inflammatory responses preceding the clinical manifestation of the leakage, rather than being a consequence of established sepsis. Consequently, these findings should be interpreted as predictive tools for delayed-onset AL, and their utility for early-onset cases requires further investigation.

In healthy individuals, HCT and ALB levels are typically stable. Infectious agents activate the monocyte-macrophage system and other inflammation-responsive cells, thus producing and releasing numerous inflammatory mediators, resulting in significant damage to the organism and increased capillary permeability. Collectively, these events elevate HCT and reduce ALB levels, thus altering their ratios. Other factors contributing to changes in the HCT-ALB ratio include the hypermetabolic state in patients with AL, which causes increased ALB consumption, intestinal dysfunction leading to the increased loss of ALB, and concurrent blood loss and anemia. Some patients with AL exhibit differences in erythrocyte pressure volume and plasma ALB levels that can be used as biomarkers to diagnose infectious diseases [20]. HCT and ALB are potential predictors of sepsis and differences in HCT-ALB ratios have significant diagnostic value for infectious diseases and are closely associated with patient prognosis [21]. Therefore, the difference in HCT-ALB ratios on different PODs represents a potential indicator for differentiating AL. Although the levels of HCT and ALB decreased after surgery in patients with AL, this reduction may not differ significantly from patients who do not have AL, thus lowering the predictive accuracy of HCT-ALB for AL.

Numerous retrospective studies report that the NAR is correlated with the severity, prognosis, and mortality rates of patients with sepsis [7]. The development of AL is a complex process. The NAR includes neutrophils and ALB levels, indicates an inflammatory response and immunotropic status, and provides a comprehensive estimate of the overall condition of an organism. Moreover, the NAR compensates for the limitations of individual inflammatory markers (e.g., CRP, PCT, leukocytes, and neutrophils) by utilizing neutrophil percentages and ALB levels, which are easily calculated from routine blood tests.

The NLR can predict AL; however, existing evidence for this relationship is inconclusive, and the optimal time point and critical values remain debatable [22]. The NLR is a predictive biomarker for symptomatic AL and may enhance the clinical possibility of new symptomatic ALs. In our comprehensive comparison, we found that HCT-ALB and NLR on POD3, and NAR and NLR on POD5, showed diagnostic and predictive value for AL. Of these factors, NAR on POD5 exhibited the highest predictive accuracy, followed by NLR on POD5. HCT-ALB and NLR on POD3 were less accurate for predicting AL.

HCT-ALB, NAR, NLR and other systemic inflammatory hematological monitoring indicators can be used to assess a patient’s inflammatory state and systemic metabolic status. AL is a complex systemic reaction resulting from interactions between the inflammatory response, immune system, nutritional status, gut microorganisms and other factors. Therefore, HCT-ALB, NAR, and NLR may all represent predictors for AL. However, HCT-ALB and NLR on POD3 are less accurate for the prediction of AL, possibly because AL is absent and the body has not yet initiated a systemic reaction. In addition, the postoperative reduction in HCT and ALB levels in patients with AL may not differ significantly. However, HCT-ALB and NLR on POD3, and NAR and NLR on POD5, demonstrated diagnostic and predictive values for AL. These indicators can supplement existing indices for diagnosing AL, improve early diagnostic rates, and enable timely interventions to improve prognoses.

This study had some limitations that need to be considered. For example, local drainage promotes the primary healing of AL, and systemic inflammatory markers undergo consequential change following local changes in fluid drainage [23]. The relatively small number of AL cases (*n* = 40) may have limited statistical power and increased the risk of overfitting in our ROC analyses. The reported AUC values (particularly the high AUC of 0.910 for NAR on POD5) are likely optimistic. Advanced variable selection methods such as LASSO regression were not employed, as the study was exploratory in nature rather than aimed at developing a finalized predictive model. Therefore, these results should be interpreted as hypothesis-generating and require validation in larger, independent cohorts before clinical application. Larger, multi-center prospective studies are now needed to validate these findings. Due to the limited number of clinical cases (*n* = 40 AL events), subgroup analyses—such as stratifying by tumor location (colon cancer vs. rectal cancer) or specific surgical techniques—were not performed. Variations in anastomotic leakage risk and underlying mechanisms across different tumor locations or surgical methods may limit the clinical applicability and precision of the findings. Furthermore, no internal validation such as bootstrapping was performed, meaning the reported diagnostic performance metrics may be over-optimistic and not fully generalizable. Also, the cut-off values reported here should be considered preliminary and require prospective validation before routine clinical use. The inflammatory markers selected in this study were obtained from peripheral blood, which changed after a delay following local fluid drainage. Therefore, testing local drainage fluid may be more beneficial for the early prediction and diagnosis of AL, with a potentially greater clinical value. Our data were collated from a single-center retrospective case-control study, thus increasing the chances of missing the diagnosis of clinical grade AL, potential selection bias and information bias. Some cases were excluded owing to an incomplete set of clinical information. Consequently, some factors that could exert influence on AL did not exhibit significant differences, including age, gender, BMI, smoking history, drinking history, hypertension history, diabetes history and tumor stage [24]. These inherent limitations of retrospective design may affect causal inference and require cautious interpretation. All data were derived from a single tertiary medical center, where patient demographics, surgical expertise, and perioperative protocols may differ from other regions or healthcare systems. External validation in multi-center or diverse populations is therefore essential before broader clinical adoption. In addition, several potentially influential but unmeasured confounders, including precise anastomotic technique, intraoperative perfusion assessment, and patient-specific microcirculatory status, were not recorded and may have contributed to residual confounding. In future efforts, the sample size should be increased to enhance credibility and enable the development of an accurate prediction model for AL. Furthermore, our initial statistical analysis of postoperative repeated measures data using independent t-tests or rank-sum tests at each time point failed to account for within-subject correlations and group-by-time interactions, potentially leading to the misinterpretation of temporal trends in AL prediction.

In addition, the retrospective nature and the limited number of anastomotic leakage (AL) events (*n* = 40) restricted our capability to eliminate the risk of model overfitting or potential causal inversion for late-stage biomarkers. Although separate temporal assessments were consistent and our primary multivariable model focused on the POD 3 window to avoid severe collinearity, the inflated odds ratios and wide confidence intervals for certain indices must be interpreted with caution. These findings should be regarded as exploratory chronological trends, and future large-scale prospective cohort studies are warranted to rigorously validate the independent prognostic weight of these parameters. In summary, future studies should prioritize: (1) external validation of these biomarkers in large, prospective, multi-center cohorts to confirm generalizability and refine cut-off values; (2) evaluation of dynamic monitoring such as serial measurements beyond POD5) to better capture temporal trends in inflammatory response; and (3) interventional trials assessing whether early actions triggered by abnormal values—such as intensified nutritional support, inflammation modulation, closer surveillance, or preemptive imaging—can reduce AL incidence, severity, or associated morbidity.

## Conclusions

In this research, we identified key hematological monitors of systemic inflammation. For example, the difference between erythrocyte pressure volume (HCT) and serum albumin (ALB) (HCT-ALB), the neutrophil-to-albumin ratio (NAR), and the neutrophil-to-lymphocyte ratio (NLR), were found to be more reflective of systemic condition than inflammatory markers alone when predicting Al. Tumor location (rectum), HCT-ALB, NLR on POD3, and NAR and NLR on POD5, were all identified as associate with AL after surgery for colon cancer laparoscopy. During the postoperative recovery of patients with CRC, clinicians should monitor for AL when the tumor is located in the rectum, HCT-ALB is ≥ 5.8 and NLR is ≥ 7.75 on POD3, or when NAR is ≥ 2.09 and NLR is ≥ 7.19 on POD5. HCT-ALB, NAR, and NLR are composite indices derived from routine, low-cost, and readily available laboratory tests, requiring no additional assays. Their ease of calculation and rapid availability position them as practical early-warning tools for postoperative monitoring in resource-limited settings.

## Data Availability

The datasets generated and/or analyzed during the current study are available from the corresponding author on reasonable request.
